# The Bidirectional Relationship Between Cancer Epigenetics and Metabolism

**DOI:** 10.1146/annurev-cancerbio-070820-035832

**Published:** 2020-11-30

**Authors:** Luke T. Izzo, Hayley C. Affronti, Kathryn E. Wellen

**Affiliations:** Department of Cancer Biology and Abramson Family Cancer Research Institute, Perelman School of Medicine, University of Pennsylvania, Philadelphia, Pennsylvania 19104, USA

**Keywords:** cell metabolism, epigenetics, chromatin modification, cancer

## Abstract

Metabolic and epigenetic reprogramming are characteristics of cancer cells that, in many cases, are linked. Oncogenic signaling, diet, and tumor microenvironment each influence the availability of metabolites that are substrates or inhibitors of epigenetic enzymes. Reciprocally, altered expression or activity of chromatin-modifying enzymes can exert direct and indirect effects on cellular metabolism. In this article, we discuss the bidirectional relationship between epigenetics and metabolism in cancer. First, we focus on epigenetic control of metabolism, highlighting evidence that alterations in histone modifications, chromatin remodeling, or the enhancer landscape can drive metabolic features that support growth and proliferation. We then discuss metabolic regulation of chromatin-modifying enzymes and roles in tumor growth and progression. Throughout, we highlight proposed therapeutic and dietary interventions that leverage metabolic-epigenetic cross talk and have the potential to improve cancer therapy.

## INTRODUCTION

**Figure 1:**
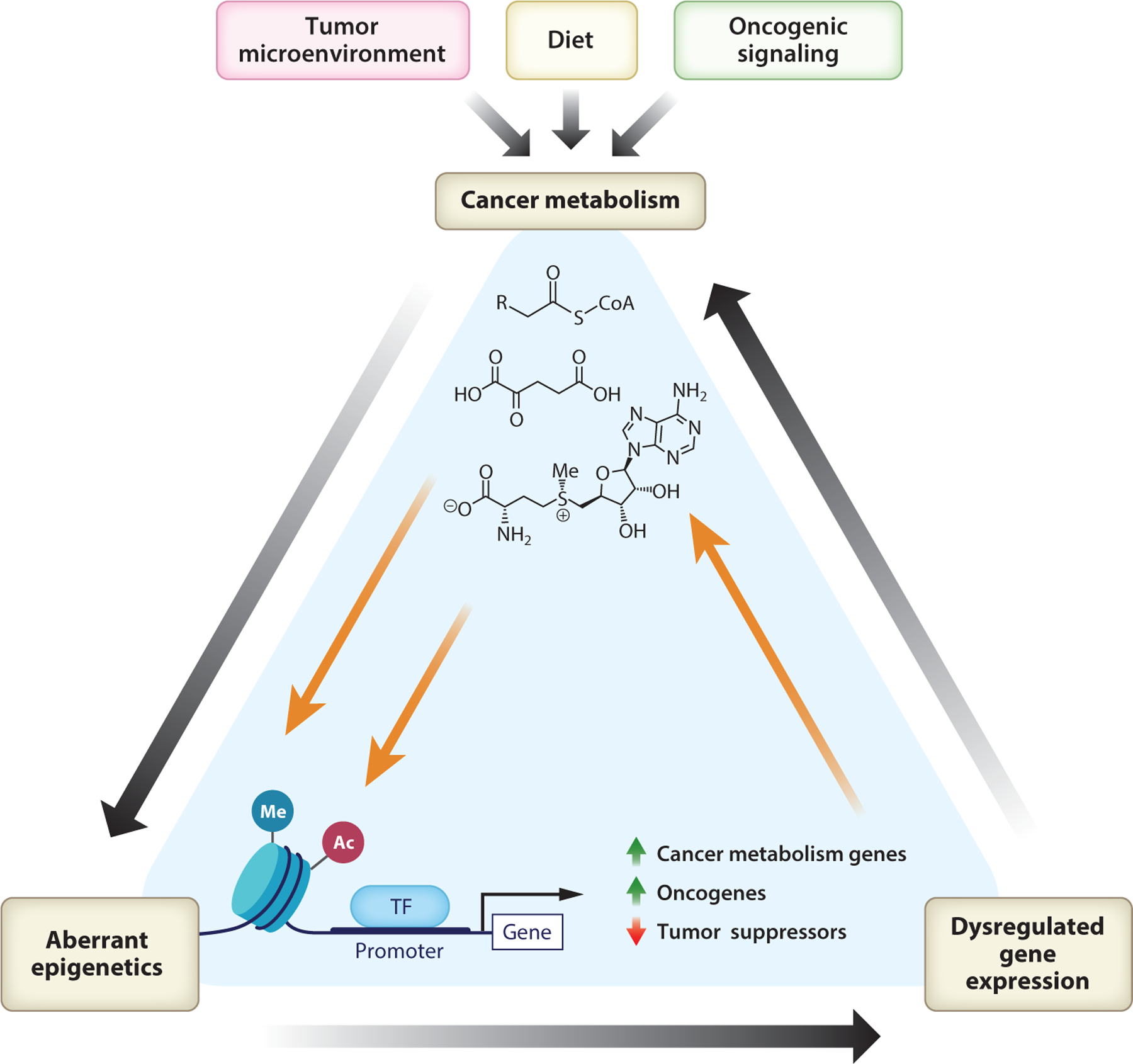
Metabolic and epigenetic reprogramming in cancer cells exert reciprocal regulation on one another. The tumor microenvironment, oncogenic signaling, and systemic metabolism, including the individual’s diet, each influence the availability of metabolites utilized by epigenetic enzymes. Tumor epigenetic features can reciprocally drive changes in the expression of genes that impact cancer metabolism. Figure adapted from images created in Biorender. Abbreviations: Ac, acetylation; Me, methylation; TF, transcription factor.

The development and progression of cancer involves the acquisition of several hallmark features, including altered metabolism (Hanahan & Weinberg 2011). Both genetic and epigenetic mechanisms can contribute to metabolic reprogramming in cancer cells. Reciprocally, ample evidence identifies metabolite abundance as a relevant factor in regulating the tumor epigenome, highlighting the substantial bidirectional cross talk between cellular metabolism and epigenetics in the context of cancer cell biology (Figure 1). Understanding the metabolism-epigenetics cross talk in distinct cancer types and how it is influenced by dietary and tumor microenvironmental factors may help to identify context-specific targetable vulnerabilities. The goals of this article are to review the mechanisms that link metabolic and epigenetic reprogramming in cancer cells and to discuss possible therapeutic strategies leveraging this interplay.

## ROLES OF EPIGENETIC ALTERATIONS IN MEDIATING METABOLIC REPROGRAMMING

Metabolic reprogramming occurs essentially universally in malignancy, although different tumors have distinct metabolic characteristics, driven by their genetic and epigenetic features, microenvironment, and cell of origin (Pavlova & Thompson 2016, Vander Heiden & DeBerardinis 2017). Epigenetic reprogramming is widespread in tumors, with mutations in genes encoding epigenetic regulators found in roughly 50% of human cancers. Even tumors without such mutations exhibit altered DNA or histone modification profiles, coinciding with changes in expression or activity of chromatin modifiers. Epigenetic alterations may result in more restrictive or permissive chromatin, modulating cellular capacity to differentiate or adapt (Flavahan et al. 2017). Here, we discuss roles of the tumor epigenome in mediating metabolic alterations, focusing on potential metabolic vulnerabilities resulting from tumor epigenetic features (Table 1).

### Histone Methylation

Histone methylation is regulated by histone methyltransferases and demethylases that add and remove histone methyl marks, respectively. In this section, we discuss evidence that mutations in or overexpression of histone methyltransferases or demethylases mediates metabolic reprogramming in cancer cells, focusing on enhancer of zeste homolog 2 (EZH2), euchromatic histone lysine methyltransferase 2 (EHMT2), lysine methyltransferase 2D (KMT2D), and lysine-specific histone demethylase 1 (LSD1).

**Figure 2:**
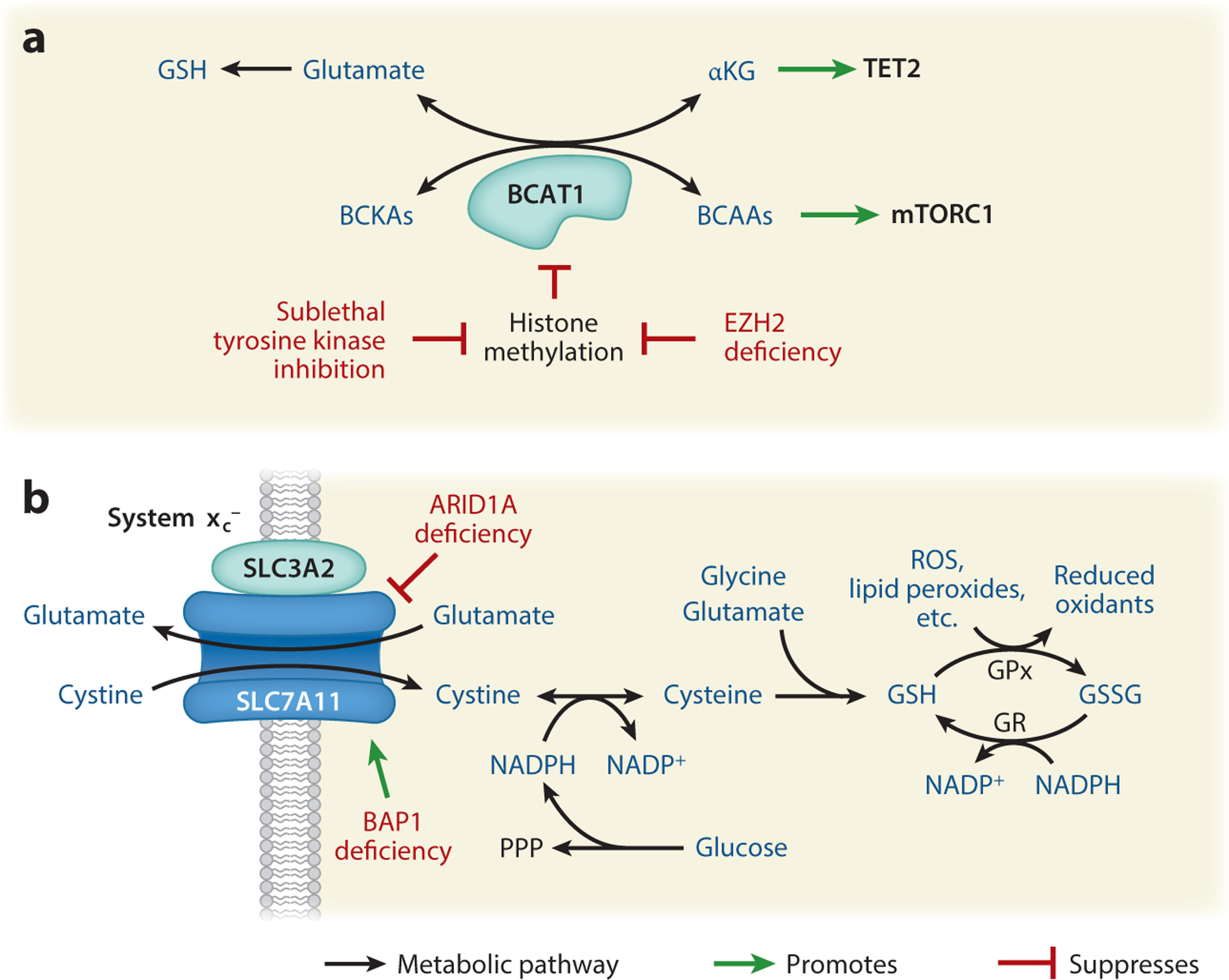
Deficiency in epigenetic enzymes alters expression of metabolic genes. (*a*) BCAT1 expression is suppressed by histone methylation. Loss of repressive histone methylation occurs with EZH2 deficiency, as well as some cancers treated with sublethal tyrosine kinase inhibition. BCAT1 catalyzes the reversible transamination of BCAAs to BCKAs using αKG as an amino group acceptor and glutamate as an amino group donor. The substrates and products of the reaction catalyzed by BCAT1 impact the generation of downstream metabolites such as GSH and impinge on TET2 and mTORC1 activity. (*b*) The system x_c_^−^ cysteine-glutamate antiporter is a dimer of SLC7A11 and SLC3A2. Expression levels of SLC7A11 are regulated by ARID1A and BAP1. System x_c_^−^ transports intracellular cystine, which is needed to synthesize glutathione. Figure adapted from images created in Biorender. Abbreviations: αKG, alpha-ketoglutarate; BCAAs, branched-chain amino acids; BCKAs, branched-chain alpha-keto acids; GPx, glutathione peroxidase; GR, glutathione reductase; GSH, reduced glutathione; GSSG,oxidized glutathione; PPP, pentose phosphate pathway; ROS, reactive oxygen species.

Loss of metabolic gene silencing mediated by repressive histone methylation represents one mechanism through which epigenetic alterations can remodel metabolism in cancer cells. This is exemplified by aberrant expression of branched-chain amino acid transaminase 1 (BCAT1) (Gu et al. 2019, Wang et al. 2019), an enzyme that catalyzes the reversible interconversion of branched-chain amino acids (BCAAs) (leucine, isoleucine, and valine) and branched-chain alpha-keto acids (BCKAs), utilizing alpha-ketoglutarate (αKG) and glutamate as the amino group acceptor or donor, respectively (Figure 2). In NRAS^G12D^-mutant myeloproliferative neoplasms, *EZH2* deficiency is associated with elevated expression of BCAT1 (Gu et al. 2019). EZH2 is the catalytic subunit of polycomb repressive complex 2 (PRC2), which methylates lysine 27 of histone H3 (H3K27) (Comet et al. 2016), a mark associated with transcriptional repression. Increased BCAT1 expression in this context drives BCKA consumption and elevates intracellular BCAA levels, potentiating the mTORC1 signaling pathway. Critically, inhibition of either BCAT1 or mTOR is selectively detrimental to *EZH2-*null cells and prevents the transition of myeloproliferative neoplasms to leukemia (Gu et al. 2019). Similarly, loss of H3K9 methylation at the *BCAT1* gene promoter coincides with increased *BCAT1* expression in tyrosine kinase inhibitor (TKI)-resistant epidermal growth factor receptor (EGFR)-mutant lung cancer cells. In this case, BCAT1 predominantly consumes BCAAs, with concomitant production of glutamate, increasing synthesis of glutathione, a tripeptide of cysteine, glycine, and glutamate. BCAT1 inhibition or treatment with reactive oxygen species–inducing agents improves TKI sensitivity (Wang et al. 2019). Thus, derepression of *BCAT1* owing to loss of repressive histone methylation results in distinct metabolic vulnerabilities (Figure 2).

Loss of function of the H3K4 methyltransferase KMT2D is also frequently observed in cancers. Loss of KMT2D in lung cancer disrupts enhancer signatures genome wide, including one regulating the circadian clock gene period circadian regulator 2 (*PER2*). Downregulation of PER2 due to the loss of KMT2D increases the expression of glycolysis genes and confers sensitivity to the glycolysis inhibitor 2-deoxyglucose (Alam et al. 2020). KMT2D repression in pancreatic cancer cells similarly promotes glycolytic gene expression (Koutsioumpa et al. 2019). KMT2D protein level is also regulated by the ubiquitin E3 ligase FBXW7, with loss of KMT2D promoting growth of diffuse large B cell lymphoma cells. FBXW7 deficiency increases KMT2D stability, leading to suppression of oxidative phosphorylation genes and sensitizing cells to mitochondrial inhibition (Saffie et al. 2020).

**Figure 3:**
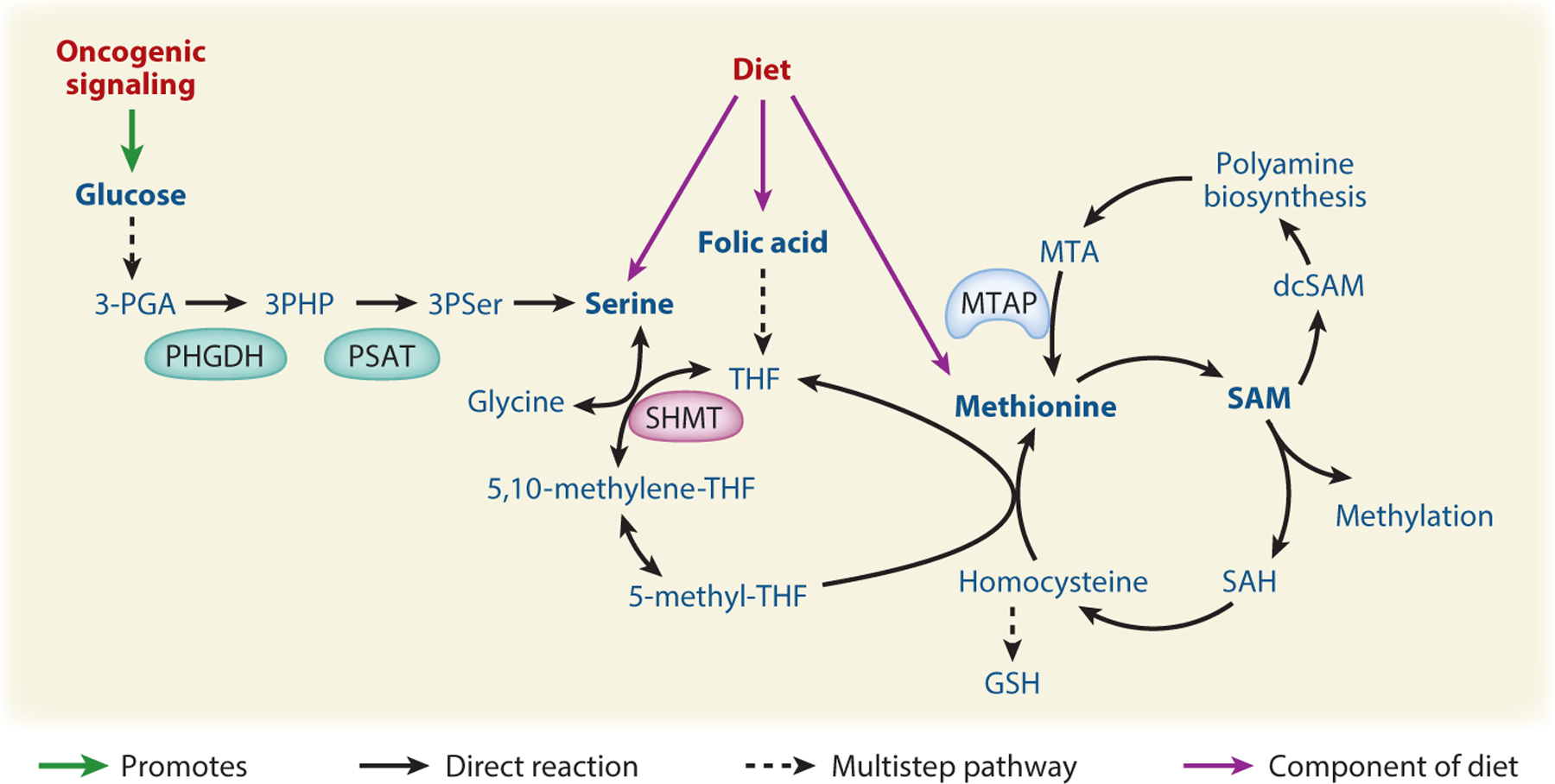
Oncogenic signaling and diet impact one-carbon metabolism and methylation. One-carbon metabolism is composed of folate metabolism and the methionine cycle and is important for DNA synthesis and the SAM production needed for methylation reactions. Dietary availability of serine, folate, and methionine, as well as oncogenic signaling and microenvironmental nutrient availability, can impact the serine-glycine one-carbon network, leading to epigenetic alterations and exposing therapeutic vulnerabilities. Figure adapted from images created in Biorender. Abbreviations: 3-PGA, 3-phosphoglyceric acid; 3PHP, 3-phosphohydroxypyruvate; 3PSer, 3-phosphoserine; dcSAM, decarboxylated SAM; GSH, glutathione; MTA, methylthioadenosine; MTAP, MTA phosphorylase; PHGDH, phosphoglycerate dehydrogenase; PSAT, phosphoserine aminotransferase; SAH, *S*-adenosyl homocysteine; SAM, *S*-adenosyl methionine; SHMT, serine hydroxymethyltransferase; THF, tetrahydrofolate.

Elevated expression of the H3K9 mono- and dimethyltransferase EHMT2 occurs in several cancers (Casciello et al. 2015). Transcriptomic analysis of non-small-cell lung cancer (NSCLC) cell lines upon EHMT2 inhibition revealed its role in regulating the serine-glycine synthesis pathway. EHMT2 inhibition suppresses H3K9me1 at relevant gene promoters, including *PSAT1* (phosphoserine aminotransferase 1) and *PHGDH* (D-3-phosphoglycerate dehydrogenase), reducing expression of these genes (Figure 3). The serine-glycine synthesis pathway is required for EHMT2-dependent proliferation (Ding et al. 2013). Interestingly, H3K9me1 mediated by EHMT1/2 is implicated in maintenance of heterochromatin when exogenous methionine is limited (Haws et al. 2020), although the implications of this mechanism for tumorigenesis are not yet known.

The H3K4 demethylase LSD1 is overexpressed in hepatocellular carcinoma (HCC), and *LSD1* silencing suppresses xenograft tumor growth. The data suggest that LSD1 supports dependence on glycolysis in HCC cells, at least in part through reducing H3K4 methylation, a mark of active gene transcription, at genes associated with mitochondrial metabolism (Sakamoto et al. 2015).

### Histone Acetylation

Deregulation of histone acetylation is also frequently observed in cancer. Acetylation is regulated by histone acetyltransferases (HATs), which deposit the acetyl marks, while histone deacetylases (HDACs) remove them. Bromodomains and YEATS domains are readers of acetyl-lysine. HATs and HDACs are mutated and aberrantly expressed in cancer, but in most cases the impact of alterations in their expression and activity on cellular metabolism has been little studied (Attar & Kurdistani 2017, Han et al. 2019). One key exception is Sirtuin 6 (SIRT6), a deacetylase and tumor suppressor with roles in metabolic regulation, discussed below. Additionally, bromodomain and extraterminal motif (BET) inhibitors, which target proteins that recognize histone acetylation, have become promising therapies (Stathis & Bertoni 2018). Effects of these drugs on cellular metabolism have been identified, illuminating the potential to combine metabolic inhibitors with BET inhibitors.

SIRT6 is a potent tumor suppressor that is downregulated in nearly 20% of all human cancers (Sebastián et al. 2012). High SIRT6 expression is predictive of better survival in human pancreatic and colon cancers (Sebastián et al. 2012, Lin et al. 2013, Kugel et al. 2016), and SIRT6 deficiency enhances tumorigenesis in vivo in mice (Sebastián et al. 2012). SIRT6 serves as a corepressor for transcription factors including hypoxia-inducible factor 1-alpha (HIF-1α) and MYC, and its loss enhances expression of glycolysis genes including glucose transporter 1 (*GLUT1*), phosphor-fructokinase 1 (*PFK1*), pyruvate dehydrogenase kinase 1 (*PDK1*), and lactate dehydrogenase A (*LDHA*) (Zhong et al. 2010, Sebastián et al. 2012). PDK1 phosphorylates and inhibits pyruvate dehydrogenase, inhibiting pyruvate entry into the mitochondrial TCA (tricarboxylic acid) cycle and enforcing aerobic glycolysis. Silencing of *PDK1* suppresses tumor growth in the context of *SIRT6* deficiency (Sebastián et al. 2012). SIRT6 also regulates gene expression by binding RNA polymerase II (Pol II) to promote transcriptional pausing, thereby restraining expression of its target genes (Etchegaray et al. 2019).

BET bromodomain-containing proteins such as BRD4 bind acetyl-lysine to exert biological functions. MYC-driven transcription is particularly sensitive to BET inhibition (Zaware & Zhou 2019), and accordingly, the BET inhibitor JQ-1 was shown to downregulate *LDHA* in ovarian cancer (Qiu et al. 2015). Evidence also suggests that BET inhibitor efficacy may be enhanced in combination with specific metabolic inhibitors. For example, BRD4 interacts with MTHFD1, an enzyme in folate metabolism. The combination of JQ-1 and the antifolate methotrexate synergizes to slow cancer progress in a variety of models (Sdelci et al. 2019). Additionally, combining JQ1 with a statin, which targets the mevalonate pathway, suppresses pancreatic cancer cell proliferation in vitro and tumor growth in vivo (Carrer et al. 2019). While further mechanistic data are needed, these studies indicate the potential for cotargeting metabolic and epigenetic processes.

### Ubiquitination

Several enzymes involved in depositing or removing ubiquitination are characterized as tumor suppressors or oncogenes (Jeusset & McManus 2019). Recent work has shown that BRCA-associated protein 1 (BAP1), which functions as part of the polycomb repressive deubiquitinase complex that removes monoubiquitination on H2AK119, regulates SLC7A11, a subunit of the heterodimeric system x_c_^−^ cystine-glutamate antiporter. BAP1 deficiency causes an increase in H2A ubiquitination in the promoter and gene body of *SLC7A11*, promoting its expression (Zhang et al. 2018). Cystine (the oxidized dimeric form of cysteine) imported by cells can be used for glutathione synthesis, protecting cells from oxidative stress and ferroptosis, a form of cell death resulting from iron-dependent lipid peroxidation (Dixon & Stockwell 2019). Accordingly, *BAP1* mutations associate with high *SLC7A11* expression and resistance to ferroptosis induction (Zhang et al. 2018). Notably, NADPH is required to reduce imported cystine to cysteine, and tumors with high *SLC7A11* expression are sensitive to inhibition of the uptake of glucose, which supports NADPH production via the pentose phosphate pathway (Liu et al. 2020). (Figure 2).

### SWI/SNF Chromatin Remodeling Complex

SWI/SNF chromatin remodeling complexes use ATP hydrolysis to physically alter DNA-histone interactions, either shifting the location of or removing nucleosomes from DNA (Helming et al. 2014). Genes encoding SWI/SNF subunits are mutated in approximately 20% of human cancers. Alterations in two SWI/SNF components, *ARID1A* and *BRG1*, result in distinct metabolic dependencies (Wu et al. 2016, Ogiwara et al. 2019). A small-molecule screen found that *ARID1A*-deficient ovarian cancer cells exhibit increased sensitivity to inhibition of glutathione synthesis. Mechanistically, ARID1A facilitates expression of *SLC7A11*, and *ARID1A*-mutant cells are thus deficient in cystine import, resulting in sensitivity to further glutathione depletion (Ogiwara et al. 2019) (Figure 2). *BRG1* is an established tumor suppressor in several types of cancer but is overexpressed in some breast cancers. In BRG1-overexpressing breast cancer cells, knockdown of BRG1 suppresses expression of lipogenesis genes and moderately sensitizes them to fatty acid synthesis inhibition (Wu et al. 2016).

### Enhancer Regulation

Acquisition of cancer-specific enhancers may also drive changes in expression of metabolic genes. Enhancers are DNA segments that interact with linked gene promoters to stimulate transcription. Superenhancers (SEs) are large clusters of enhancers that typically drive cell identity and disease-related gene expression. These regions are enriched in H3K27ac, are sensitive to BET inhibition, and are typically occupied by numerous transcription factors, cofactors, enhancer-associated proteins and RNAs, signaling factors, and Pol II (Bradner et al. 2017).

Recent studies have identified roles for oncogenic SEs in metabolic gene regulation (Nguyen et al. 2015, Gimple et al. 2019, Tsang et al. 2019). Interrogation of H3K27ac ChIP-seq (chromatin immunoprecipitation followed by sequencing) data sets identified SE regions enriched in glioma stem cells (GSCs). A GSC-specific SE regulates the expression of elongation of very long-chain fatty acids protein 2 (ELOVL2), an endoplasmic reticulum transmembrane protein that functions in long-chain polyunsaturated fatty acid (LC-PUFA) metabolism. In GSCs, PUFAs promote EGFR signaling, and inhibition of LC-PUFA metabolism suppresses EGFR signaling and GSC growth (Gimple et al. 2019). Similarly, analysis of publicly available H3K27ac ChIP-seq data sets comparing the SE landscapes in liver cancer cell lines to normal liver tissues identified that sphingosine kinase 1 (*SPHK1*) acquires an SE in liver cancer cell lines. SPHK1 converts sphingosine to sphingosine-1-phosphate, a signaling molecule that can promote cell survival. *SPHK1* mRNA is upregulated in human HCC, and its expression negatively correlates with survival. Silencing of SPHK1 attenuates liver cancer xenograft tumor growth and metastasis (Tsang et al. 2019). Finally, endocrine therapy–resistant breast cancer cells acquire SEs at cholesterol synthesis genes, such as CYP27A1 (Nguyen et al. 2015). CYP27A1 generates 27 hydroxycholesterol (27HC) from cholesterol. 27HC can act as a modulator of the estrogen receptor (ER) (Warner & Gustafsson 2014), enabling estrogen-independent ER binding to regulatory regions. Importantly, targeting of cholesterol biosynthesis with statins suppresses ERα binding and cell invasion (Nguyen et al. 2015). These data together indicate that SEs acquired during tumorigenesis or the development of therapeutic resistance can contribute to oncogenic phenotypes in part through regulation of metabolism genes.

## METABOLIC REWIRING IN CANCER IMPACTS THE TUMOR EPIGENOME

In addition to epigenetic alterations driving metabolic reprogramming, metabolites reciprocally influence chromatin modification through their use as substrates or inhibitors of epigenetic enzymes. Cancer cell metabolism is influenced by cell-intrinsic (oncogenic signaling and genetic and epigenetic features) and -extrinsic (diet, exogenous growth factors, tumor microenvironment) factors, each of which has potential to impact metabolic regulation of the epigenome (Figure 1). In this section, we discuss how alterations in cellular metabolism or nutrient availability impact the cancer cell epigenome, with an emphasis on resultant vulnerabilities.

### Metabolic Regulation of 2-Oxoglutarate-Dependent Dioxygenases

2-Oxoglutarate-dependent dioxygenases catalyze hydroxylation reactions utilizing iron (Fe^2+^) as a cofactor and molecular oxygen and αKG (2-oxoglutarate) as cosubstrates (Islam et al. 2018) and have emerged as highly sensitive to metabolic regulation in cancer. Within this family of enzymes, the tumor suppressor TET2 oxidizes 5-methylcytosine (5-mC) to 5-hydroxymethylcytosine (5-hmC), and subsequently to 5-formylcytosine and 5-carboxylcytosine, ultimately facilitating DNA demethylation (Kohli & Zhang 2013). Lysine demethylases (KDMs) of the Jumonji-C domain–containing protein family hydroxylate methylated lysines to hydroxymethyl intermediates, mediating histone demethylation with the release of formaldehyde (Mosammaparast & Shi 2010). Metabolites with chemical structures similar to αKG, including d-2-hydroxyglutarate (d-2-HG) and l-2-hydroxyglutarate (l-2-HG), succinate, and fumarate, can competitively inhibit these enzymes, rendering them sensitive to shifts in the balance of these metabolites in cells.

#### d-2-hydroxyglutarate.

Isocitrate dehydrogenase 1 and 2 (IDH1/2) catalyze the interconversion of isocitrate and αKG. *IDH1* and *IDH2* are frequently mutated in cancer, and the IDH1/2-mutant (IDHm) enzymes acquire a neomorphic activity that results in the conversion of αKG to d-2-HG (Dang et al. 2009, Ward et al. 2010). d-2-HG has been described as an oncometabolite and has been shown to inhibit 2-oxoglutarate–dependent dioxygenases, including the TET enzymes (Golub et al. 2019). Extensive work, reviewed previously (Losman & Kaelin 2013, Raineri & Mellor 2018), has revealed that elevated d-2-HG as a consequence of IDH1/2 mutation drives aberrant DNA and histone methylation. Currently, there are two FDA (US Food and Drug Administration)–approved IDHm inhibitors (enasidenib, an inhibitor of IDH2m, and ivosidenib, an IDH1m inhibitor), with several other compounds under investigation (Golub et al. 2019).

In addition to direct targeting of the mutant IDH enzymes, IDHm tumors have distinct vulnerabilities based on their metabolic and epigenetic characteristics (Park & Turcan 2019, Stuani et al. 2019). Glutamine feeds into d-2-HG pools, and thus glutaminase (GLS) inhibitors have been investigated for IDHm cancers (Seltzer et al. 2010, Emadi et al. 2014). d-2-HG also inhibits αKG use by BCAT1 and BCAT2, rendering IDHm cells deficient in glutamate and, consequently, glutathione production (Figure 2). IDHm cells are thus reliant on glutamate synthesis from glutamine and are particularly sensitive to GLS inhibition in combination with oxidative stress–inducing radiation, revealing a unique therapeutic point of leverage for treating IDHm cancers (McBrayer et al. 2018). The clinical strategy of targeting GLS in combination with radiation and temozolomide is currently being tested in a clinical trial for IDHm diffuse or anaplastic astrocytoma (https://clinicaltrials.gov/ identifier NCT03528642).

#### l-2-hydroxyglutarate.

The l enantiomer of 2HG, l-2-HG, is also elevated in some cancers, due to either loss of l-2-HG dehydrogenase (L2HGDH) or microenvironmental conditions including hypoxia and acidity. L2HGDH deficiency leads to l-2-HG accumulation, which drives loss of 5-hmC and aberrant DNA methylation in clear cell renal cell carcinoma (ccRCC) (Creighton et al. 2013, Hu et al. 2014, Shim et al. 2014, Shenoy et al. 2015). l-2-HG production also increases in hypoxia due to the promiscuous activity of lactate and malate dehydrogenase enzymes (Intlekofer et al. 2015, Oldham et al. 2015), which is promoted by acidic pH (Intlekofer et al. 2017). Hypoxia-induced l-2-HG accumulation inhibits αKG–dependent enzymes, resulting in elevated histone methylation, as well as HIF-1α accumulation, thereby potentiating the hypoxic response (Intlekofer et al. 2015, 2017).

#### Succinate and fumarate.

Fumarate hydratase (FH) or succinate dehydrogenase (SDH) loss-of-function mutations lead to a buildup of fumarate or succinate, respectively, and are found in paragangliomas, pheochromocytomas, leiomyomatosis, and renal cell cancer (Favier et al. 2015, Yong et al. 2020). The accumulation of succinate resulting from SDH mutations inhibits αKG-dependent HIF prolyl hydroxylases, resulting in stabilization of HIF1 and HIF2 (Selak et al. 2005). Accumulation of succinate in SDH-null cells also inhibits TET enzymes, leading to DNA hypermethylation (Letouzé et al. 2013). Notably, combined activation of HIF2 and inhibition of TET enzymes mimics the metastatic phenotype seen in SDHB-mutant cells (Morin et al. 2020). Inactive FH in renal cancer cells promotes fumarate accumulation, reducing TET enzyme activity, leading to increased DNA methylation. DNA methylation occurs at regulatory regions controlling the antimetastatic mir-200 cluster, decreasing its expression and increasing expression of epithelial-mesenchymal transition (EMT)-related genes (Sciacovelli et al. 2016).

#### α-Ketoglutarate.

In addition to aberrant accumulation of inhibitory metabolites, the production of the TET cosubstrate αKG itself is a point of regulation of TET enzymes in cancer cells. Tumor suppression by p53 in a mouse model of Kras^G12D^-driven pancreatic cancer depends on control of the αKG:succinate ratio (Morris et al. 2019). Using a model with doxycycline-inducible p53 expression, Morris et al. showed that re-expression of p53 after tumor formation triggers differentiation and tumor suppression. Intriguingly, this is associated with an increase in the αKG:succinate ratio, along with elevated 5-hmC levels, consistent with an increase in TET activity. Remarkably, manipulation of enzymes that modulate αKG mirrors p53 reactivation. Silencing of oxoglutarate dehydrogenase (OGDH), the enzyme that converts αKG to succinyl-CoA (coenzyme A), increases the αKG:succinate ratio, enhances 5-hmC levels, and reduces tumor growth in p53-null tumors in vivo (Morris et al. 2019).

αKG is also produced or consumed in the cell in several transaminase reactions, and recent evidence suggests that transaminases such as BCAT1 may also sufficiently impact αKG availability to modulate TET enzyme activity. Proteomic analysis in acute myeloid leukemia (AML) found enrichment for BCAT1 in leukemia stem cells. BCAT1 knockdown increases αKG levels, increasing TET activity and impairing cell growth and colony-formation in cells derived from AML patient samples. Notably, high BCAT1 expression inversely correlates with survival only in AML patients whose cancers were wild-type for TET2 and IDH, consistent with BCAT1 overexpression promoting tumorigenesis via suppression of TET2 function (Raffel et al. 2017).

αKG availability is further subject to constraints dictated by the tumor microenvironment. Low glutamine availability in the tumor microenvironment limits αKG levels and the activity of 2-oxoglutarate–dependent dioxygenases, resulting in hyper histone methylation within the interior of melanoma tumors (Pan et al. 2016). Glutamine-deprived melanoma cells are resistant to BRAF inhibitors but can be sensitized by inhibition of the methyltransferase EZH2 (Pan et al. 2016), pointing to a direct mechanism through which microenvironmental nutrient gradients induce therapeutic resistance.

In the lethal pediatric malignancy posterior fossa A (PFA) ependymoma, hypoxia plays a key role in driving metabolic and epigenetic reprogramming and is required for the maintenance of PFA ependymoma cells in culture. H3K27me2 and me3 are suppressed under hypoxia, enforced by a high αKG:succinate ratio, which promotes the activity of the H3K27 demethylases KDM6A and 6B. PFA ependymoma cells are sensitive to inhibition of these demethylases as well as inhibition of GLS, which feeds αKG pools. Increased availability of the methyl donor SAM in these cells can similarly drive an increase in H3K27 methylation and suppress PFA growth (Michealraj et al. 2020).

#### Metabolic strategies to enhance TET activity.

With accumulating evidence that impairment of TET2 function may be a major mechanism through which metabolic reprogramming promotes cancer progression, metabolic strategies to enhance TET activity have been investigated. These strategies include metabolic manipulations to boost αKG levels, stimulation of TET activity through ascorbate supplementation, and enhancement of the stability of TET2 via AMPK-dependent phosphorylation. Increasing αKG through inhibition of OGDH increased TET enzyme activity and 5-hmC levels in breast tumors and inhibited metastatic spread (Atlante et al. 2018). In vivo administration of membrane-permeable dimethyl-αKG promoted cellular differentiation and suppressed tumor growth in a mouse model of colorectal cancer (Tran et al. 2020). Additionally, ascorbate is a cofactor of 2-oxoglutarate-dependent dioxygenases, and its supplementation promotes TET enzyme activity to exert anticancer effects (Agathocleous et al. 2017; Cimmino et al. 2017; Shenoy et al. 2017, 2019; Mingay et al. 2018; Mustafi et al. 2018). Finally, metabolic control of posttranslational modification of TET2 is also reported. AMPK-dependent phosphorylation of TET2 results in its stabilization. Intriguingly, hyperglycemia suppresses AMPK activity and destabilizes TET2, providing a possible mechanism linking diabetes and cancer. Metformin, a diabetes drug that promotes AMPK activation, slows growth of xenografted tumors in a TET2-dependent manner (Wu et al. 2018a).

### Metabolic Regulation of Methyltransferases

Methyltransferases can act either on nucleic acids, methylating cytosine to generate 5-mC, or on lysine and arginine residues within proteins such as histones. All methyltransferase enzymes utilize *S*-adenosyl-l-methionine (SAM) as the methyl donor, and the abundance of SAM can impact DNA and histone methylation (Reid et al. 2017, Campbell & Wellen 2018). SAM is produced within the methionine cycle from methionine and ATP (Figure 3). After SAM is used for methylation, the product *S*-adenosylhomocysteine (SAH) can be recycled to regenerate methionine and SAM via acquisition of a methyl-group provided by 5-methyl THF (tetrahydrofolate) from the integrated folate cycle and serine-glycine metabolism. The serine-glycine biosynthesis pathway branches off from glycolysis at 3-phosphoglycerate. Serine is synthesized in three steps requiring the enzymes PHGDH, PSAT, and phosphoserine phosphatase. Glycine is produced from serine by serine hydroxymethyltransferase (SHMT) enzymes, which carry out the reversible transfer of a one-carbon unit from serine to THF (Figure 3). Additionally, SAM can be decarboxylated and used for polyamine biosynthesis, which generates the by-product methylthioadenosine (MTA). MTA can be recycled through the methionine salvage pathway, for which the rate-limiting enzyme is MTA phosphorylase (MTAP), to regenerate methionine (Avila et al. 2004) (Figure 3). Here, we discuss how oncogenic and microenvironmental cues, changes in metabolic enzyme expression, and the availability of dietary methionine or folate can impact SAM-dependent histone and DNA methylation. Potential therapeutic targets that leverage this metabolic-epigenetic cross talk are highlighted.

#### Oncogenic signaling and drug resistance.

Activation of the serine-glycine one-carbon network in response to oncogenic signaling in cancer cells is linked to regulation of DNA methylation. In a pancreatic cancer context, cells expressing oncogenic KRAS and lacking the tumor suppressor LKB1 (referred to as KL cells) exhibit increased flux through the serine-glycine arm of one-carbon metabolism, promoting SAM synthesis and elevated DNA methylation. KL tumors become reliant on these metabolic and epigenetic features, and either silencing of PSAT or DNMT inhibition suppresses KL tumor growth in mice (Kottakis et al. 2016). Similarly, reduced expression of the atypical protein kinase PKCλ/*ι* is characteristic of neuroendocrine prostate cancer (NEPC), and murine prostate tumors lacking PKCλ/*ι* exhibit transcriptional upregulation of the serine-glycine biosynthesis pathway, mediated via mTORC1-dependent activation of the transcription factor ATF4. SAM abundance is elevated in PKCλ/*ι*-deficient tumors, resulting in DNA hypermethylation and NEPC differentiation. DNMT inhibition or silencing of PHGDH suppresses the growth of tumors lacking PKCλ/*ι* (Reina-Campos et al. 2019). Serine biosynthesis pathway enzymes including PHGDH and PSAT1 are upregulated in Burkitt’s lymphoma as a result of upregulated MYC/ATF4-controlled transcription. Inhibition of PHGDH decreases DNA and histone methylation, although it did not alter tumor growth (Białopiotrowicz et al. 2020). Thus, in several distinct cancer types, activation of serine-glycine biosynthesis promotes SAM synthesis and DNA hypermethylation, which in some contexts results in enhanced sensitivity to DNA methyltransferase inhibitors.

Chemotherapy-resistant breast cancer cells conversely exhibit low abundance of methionine cycle intermediates, including SAM. This corresponds with reduced DNA methylation and genome-wide compensatory reprogramming of H3K27me3, rendering cells sensitive to inhibition of the H3K27 methyltransferase EZH2 (Deblois et al. 2020).

#### Metabolic gene expression changes.

Changes in the expression of metabolic enzymes also drive changes in one-carbon metabolism and methylation in tumors. A prime example is *MTAP*, the rate limiting enzyme in the methionine salvage pathway (Figure 3), which is deleted in a variety of cancers along with the neighboring tumor suppressors *CDKN2A* and *ARF* (Mavrakis et al. 2016). *MTAP*-inactivating mutations are also associated with an autosomal-dominant bone dysplasia and cancer syndrome in humans, and MTAP heterozygous knockout in mice is sufficient to cause lymphoma, indicating that MTAP itself is a tumor suppressor (Kadariya et al. 2009, Camacho-Vanegas et al. 2012). Accordingly, in glioblastoma, MTAP-null cells exhibit enhanced expression of stemness genes and GSC formation, linked to DNA hypomethylation (Hansen et al. 2019). In addition, MTAP deficiency results in distinct vulnerabilities owing to MTAP’s metabolic role in recycling the polyamine metabolic product MTA. MTAP deficiency leads to a buildup of MTA, which can compete with SAM for binding to the type II (symmetric) arginine methyltransferase PRMT5, reducing histone arginine methylation. The resultant low-PRMT5 activity renders MTAP-deficient cancer cells highly sensitive to further PRMT5 inhibition (Kryukov et al. 2016, Marjon et al. 2016, Mavrakis et al. 2016). Interestingly, combined PRMT5 and type I (asymmetric) PRMT inhibition exhibit combinatorial effects in blocking cancer cell proliferation and xenograft tumor growth. MTAP-deficient cells are thus also more sensitive to type I PRMT inhibition (Fedoriw et al. 2019, Gao et al. 2019a). Altogether these data indicate that MTAP loss elicits widespread epigenetic changes that both contribute to tumorigenesis and create targetable epigenetic dependencies.

Nicotinamide N-methyltransferase (NNMT), which methylates nicotinamide using SAM, is overexpressed in a variety of cancers including lung, liver, kidney, bladder, and colon. NNMT overexpression depletes SAM pools, promoting hypomethylation of histones and driving protumorigenic gene expression (Ulanovskaya et al. 2013). NNMT is also highly expressed in metastasis-associated cancer-associated fibroblasts (CAFs) in the context of ovarian cancer. NNMT-dependent depletion of SAM pools and histone methylation facilitates expression of CAF markers and promotes tumor growth and metastasis (Eckert et al. 2019).

Elevated IDH3α expression was recently found to promote glioblastoma growth. In contrast to IDH1/2, IDH3α, a subunit of the heterodimeric TCA cycle enzyme IDH3, is not mutated but is overexpressed in glioma, and it exerts its effects at least in part through interaction with the cytosolic SHMT (cSHMT). cSHMT is a reversible enzyme that converts THF and serine to glycine and 5,10-methylene THF (Figure 3). 5,10-methylene THF provides one-carbon units needed to regenerate methionine from homocysteine for SAM synthesis and to synthesize thymidylate needed for DNA synthesis. The cSHMT-IDH3α interaction promotes the partitioning of one-carbon units towards nucleotide synthesis during S phase and away from SAM production. IDH3α depletion promotes hypermethylation of cancer-relevant genes and reduces cancer progression, suggesting that targeting IDH3 or the interaction of cSHMT and IDH3α could hold therapeutic potential for glioblastoma (May et al. 2019).

#### Nutrition.

Folate plays a critical role in methylation and nucleotide metabolism, and thus it is studied in numerous processes from embryonic development to tumorigenesis. Nutritional epidemiology studies have tied folate consumption to cancer incidence, although the directionality appears to be highly context dependent (Pieroth et al. 2018). Studies in liver, colon, and prostate cancer also yield varying results as to the extent to which dietary folate impacts DNA methylation (Kim et al. 1996, Song et al. 2000, Kim 2005, Pogribny et al. 2006, Bistulfi et al. 2011). More recently, work in prostate cancer xenografts finds that both folate supplementation and depletion have dramatic effects on DNA methylation due to changes in the ratio of SAM to SAH. Interestingly, timing of dietary folate manipulation is important, whereby dietary folate restriction starting at the same time of androgen withdrawal therapy reduces the recurrence rate of castration-resistant prostate cancer, while folate restriction prior to xenograft implantation does not (Affronti et al. 2017). Thus, dietary folate abundance appears to have both timing- and context-dependent effects on methylation in tumors, and further work is needed to clarify optimal strategies for therapeutic dietary folate manipulation.

SAM pools and histone methylation are also impacted by dietary methionine content. Methionine restriction can reduce SAM pools and histone methylation both in vitro and in vivo, and beneficial effects of methionine restriction are reported in the contexts of aging, obesity, and cancer (Sanderson et al. 2019, Wanders et al. 2020). Methionine deprivation may be most effective as part of a combinatorial strategy; chemotherapy and radiation therapy efficacy are improved by methionine restriction in preclinical models (Gao et al. 2019b). Recent evidence also indicates that avid methionine consumption by cancer cells may promote immune evasion by reducing T cell methionine uptake and H3K79 dimethylation, which regulates STAT5 expression. Methionine supplementation or inhibition of tumor cell methionine uptake boosts T cell immunity (Bian et al. 2020).

High-fat diet, which is used to model diet-induced obesity in rodents, may also impact SAM pools and histone methylation in cancer cells. In a mouse model of MYC-driven prostate cancer, a diet high in fat promotes cancer progression via potentiation of the MYC transcriptional program (Labbé et al. 2019). MYC expression reduces SAM and increases SAH levels in prostate cancer, and these effects are exacerbated by high-fat diet. Histone methylation dynamics are altered accordingly, including pronounced hypomethylation of H4K20 globally and at the promoters of MYC target genes. Importantly, the augmented Myc signature in mice can be reversed by switching to a control diet. In addition, clinical samples from prostate cancer patients stratified by saturated fat intake reveal enrichment of a MYC target gene signature and greater overall mortality in those consuming high amounts of saturated fat. These data thus suggest that diet has the potential to impact disease progression in part by modulating the epigenome.

### Metabolic Regulation of Acetylation

Histone acetylation is associated with active gene transcription and is determined by the respective activities of acetyltransferases and deacetylates. Histone lysine acetyltransferases transfer the acetyl group from acetyl-CoA to the *ε*-amine of the lysine side chain, neutralizing the positive charge of the unmodified residue. Histone acetylation is highly sensitive to the availability of acetyl-CoA. HDACs are also subject to metabolic regulation. Sirtuin (class III) HDACs rely on nicotinamide adenine dinucleotide (NAD^+^) for their activity, while the metabolites butyrate and β-hydroxybutyrate (BHB) can inhibit class I and IIa HDACs.

#### Acetyl-CoA.

Acetyl-CoA availability for histone acetylation is determined by its production and utilization within the nuclear-cytosolic compartment. Extramitochondrial acetyl-CoA is primarily generated from citrate or acetate via ACLY and ACSS2, respectively, and abundant evidence has linked each of these enzymes to the regulation of histone acetylation in diverse contexts. Data have also emerged supporting the notion that nuclear production of acetyl-CoA by ACLY, ACSS2, or the pyruvate dehydrogenase complex (PDC) is important for processes, including transcription of specific genes and DNA damage repair. These findings and concepts are extensively covered in previous reviews (Shi & Tu 2015, Li et al. 2018, Sivanand et al. 2018). Here we focus on evidence that (*a*) oncogenic or stress-induced signaling pathways and (*b*) altered expression or localization of acetyl-CoA metabolic enzymes regulate acetyl-CoA production and histone acetylation in cancer.

##### Signaling.

Both ACLY and ACSS2 have been shown to be directly regulated by signaling cascades that respond to growth factor stimulation or nutrient availability. The regulation of these enzymes, as well as acetyl-CoA-consuming enzymes, directly impacts acetyl-CoA levels and alters histone acetylation. AKT and AMPK are two major signaling factors that have emerged as regulators of acetyl-CoA metabolism and histone acetylation. The PI3K-AKT signaling pathway promotes cell survival, growth, and proliferation and is commonly activated in cancer cells (Manning & Toker 2017). AMPK is a sensor of intracellular energy stress, which exerts broad effects to inactivate anabolic processes and stimulate nutrient uptake and catabolism (González et al. 2020). Both signaling nodes regulate acetyl-CoA levels and histone acetylation, as we discuss below.

Substantial evidence points to the phosphorylation of ACLY at serine 455, which increases its activity (Potapova et al. 2000), as a key point of control for this enzyme. Several kinases phosphorylate this site, including AKT, PKA, and BCKDK (Guy et al. 1980, Berwick et al. 2002, White et al. 2018), with AKT being the most extensively studied in recent years. Interestingly, ACLY is a mTORC2-dependent AKT substrate (i.e., dependent on AKT-S473 phosphorylation, the site phosphorylated by mTORC2) (Martinez Calejman et al. 2020).

AKT signaling is a key regulator of ACLY-S455 phosphorylation and histone acetylation in cancer cells. ACLY-S455 phosphorylation promotes global histone acetylation in cancer cells, and pAKT-S473 correlates positively with histone acetylation levels in human prostate tumors and gliomas (Lee et al. 2014). Oncogenic KRAS signaling in murine pancreatic acinar cells also promotes elevated histone acetylation in an AKT- and ACLY-dependent manner, even prior to tumor formation, and genetic deletion of *Acly* suppresses pancreatic carcinogenesis (Carrer et al. 2019). Insulin signaling, which activates the PI3K-AKT pathway, can also drive an increase in histone acetylation in cancer cells (Carrer et al. 2019, Senapati et al. 2019). In addition to oncogenic signaling, DNA damage signaling also promotes ACLY-S473 phosphorylation within the nucleus in an ATM- and AKT-dependent manner. ACLY facilitates histone H4 acetylation near sites of DNA double-strand breaks, promoting DNA repair by homologous recombination (HR). Consistent with a role in HR, ACLY silencing sensitizes cells to PARP inhibition (Sivanand et al. 2017). Thus, AKT-ACLY signaling is an important determinant of histone acetylation within cancer cells, although more work is needed to understand the functional consequences and potential for therapeutic intervention.

AMP-activated protein kinase (AMPK) impacts acetyl-CoA pools and histone acetylation in cancer through phosphorylation of at least two relevant targets: acetyl-CoA carboxylase (ACC1) and ACSS2. Inhibitory phosphorylation of ACC1 increases acetyl-CoA abundance by preventing its conversion to malonyl-CoA and increases global histone acetylation (Galdieri & Vancura 2012, Galdieri et al. 2016). Activating AMPK with metformin also boosts histone acetylation and improves efficacy of the HDAC inhibitor panobinostat in a subcutaneous bladder tumor model (Okubo et al. 2019). Reciprocally, in AML cells, AMPK deficiency reduces acetyl-CoA pools and histone acetylation, decreasing BET protein recruitment to chromatin. Treatment with AMPK and BET inhibitors synergistically inhibits leukemogenesis in mice (Jiang et al. 2019). The AMPK-ACC1 axis may also contribute to obesity-linked breast cancer. Elevated leptin and TGFβ levels in the context of obesity activate AMPK-dependent ACC1 phosphorylation in breast cancer cells, increasing acetyl-CoA and protein acetylation levels including acetylation of the transcription factor SMAD2. Acetylated SMAD2 drives expression of an EMT gene expression program, along with increased migration and invasion (Rios Garcia et al. 2017). In addition to regulating acetyl-CoA pools through ACC1 phosphorylation, AMPK phosphorylates ACSS2, promoting its nuclear localization and interaction with the transcription factor TFEB to promote histone acetylation at autophagy and lysosome biogenesis genes (Li et al. 2017). Cumulatively, the data identify AMPK as a key signaling molecule in control of acetyl-CoA pools.

Finally, PDC can also provide acetyl-CoA for histone acetylation. Growth factor signaling and mitochondrial stress promote translocation of PDC to the nucleus where it can provide acetyl-CoA for histone acetylation (Sutendra et al. 2014). However, the underlying mechanisms governing PDC nuclear localization remain poorly understood.

##### Altered expression.

Aberrant expression of acetyl-CoA producers or consumers may also impact histone acetylation. ACLY and ACSS2 are both regulated by SREBP transcription factors, and expression of each is elevated in numerous cancer types (Hatzivassiliou et al. 2005, Comerford et al. 2014, Mashimo et al. 2014, Schug et al. 2015, Carrer et al. 2019). ACSS2 is also upregulated under hypoxic conditions and plays a key role in recycling HDAC-derived acetate to sustain histone acetylation (Schug et al. 2015, Bulusu et al. 2017). Additionally, a recent study reported that an unexpected source of acetyl-CoA for histone acetylation is nuclear glycogenolysis. In lung cancer cells, low expression of the E3 ubiquitin ligase malin, which targets glycogen phosphorylase to the nucleus, results in accumulation of nuclear glycogen and suppression of the contribution of glycogen to histone acetylation. Malin re-expression promotes nuclear glycogenolysis and histone acetylation, while suppressing tumor growth (Sun et al. 2019).

The cytosolic acetyl-CoA hydrolase ACOT12 has been recently identified as another regulator of acetyl-CoA pools for histone acetylation in HCC. ACOT12 is expressed in normal liver but downregulated in HCC tumors, and expression of ACOT12 correlates negatively with HCC metastasis and decreased survival. Mechanistically, it was found that accumulation of acetyl-CoA in the absence of ACOT12 promotes EMT and metastasis, corresponding with increased histone acetylation globally and at the promoter of the EMT gene *TWIST2* (Lu et al. 2019).

#### Butyrate and β-hydroxybutyrate.

In addition to aberrant acetyl-CoA levels, inhibitors of HDACs such as butyrate can also dramatically impact histone acetylation. Butyrate is a short-chain fatty acid produced by fermentation and breakdown of fiber by the gut microbiota and used by colonocytes as a primary source of oxidizable carbon. Butyrate may produce a protective effect in inhibiting the development of colorectal cancer (Wu et al. 2018b). Buildup of butyrate, resulting in HDAC inhibition, occurs when colonocytes transform into cancerous cells as their metabolism shifts from the oxidation of butyrate to aerobic glycolysis (Donohoe et al. 2012). In studies using gnotobiotic mouse models colonized by bacteria that do or do not produce butyrate, a high-fiber diet protects animals from colorectal cancer in a manner dependent on butyrate production. Butyrate triggers elevated histone acetylation and alters gene expression in cancer cells to promote cell death and suppress proliferation (Donohoe et al. 2014). Recent work has also identified specific butyrate-producing bacteria that are suppressed in the presence of tumors and exert antiproliferative effects (Zagato et al. 2020). Notably, short-chain fatty acids produced by microbiota may impact histone modifications even outside of the colon, including in liver and adipose tissue (Krautkramer et al. 2016). More work is needed to optimize dietary and pharmacological strategies to leverage butyrate production by the microbiota in the context of cancer treatment.

BHB, a ketone body, was more recently identified as an endogenously produced inhibitor of HDACs. Serum levels of BHB can rise to low-millimolar concentrations during extended fasting or through consumption of a ketogenic diet. Although less potent than butyrate in inhibiting HDACs, BHB administration to mice increases histone acetylation and impacts gene expression in tissues (Shimazu et al. 2013). Interestingly, BHB may also be produced locally within the tumor microenvironment. Isolation of mammary gland–derived adipocytes from mastectomy patients revealed that adipocytes secrete BHB into media ex vivo, driving increased colony formation and proliferation of breast cancer cell lines (Huang et al. 2017). The improved growth correlates with an increase in H3K9 acetylation and expression of protumorigenic genes, highlighting that metabolic cross talk among cells in the microenvironment can promote tumor enhancement through epigenetic regulation.

#### Nicotinamide adenine dinucleotide.

Nicotinamide adenine dinucleotide (NAD^+^) is a redoxactive cofactor utilized by multiple enzymes including the sirtuin class of deacetylases and PARP enzymes. NAD^+^ metabolism appears to play a role in chemoprevention, potentially via the PARP DNA damage repair enzymes. A mouse model of HCC driven by expression of Uri (unconventional prefoldin RPB5 interactor) promotes tumorigenesis through downregulation of enzymes involved in NAD^+^ synthesis, resulting in increased DNA damage. Dietary supplementation of an NAD^+^ precursor, nicotinamide riboside, prevents DNA damage and suppresses HCC formation (Tummala et al. 2014). While low NAD^+^ may contribute to mutational burden and tumor formation, reducing NAD^+^ within cancer cells may conversely trigger cell death and suppress stem cell features via effects on both SIRT1 and PARP, as has been documented with inhibition of the NAD^+^ salvage enzyme NAMPT (Thakur et al. 2012, Sharif et al. 2016, Lucena-Cacace et al. 2018). NAD^+^ metabolism therefore participates in multistep tumorigenesis, with roles in modulating both PARP and sirtuin enzyme activity.

## FUTURE PERSPECTIVES

Metabolic and epigenetic remodeling in cancer cells are interwoven, influencing one another through complex mechanisms, with some common themes emerging. One theme includes the idea that metabolic regulation of TET enzyme activity by αKG and structurally similar metabolites regulate cellular differentiation and tumorigenesis. Second, AKT and AMPK signaling pathways act to promote histone acetylation via control of acetyl-CoA production by ACLY and consumption by ACC1, respectively. Third, distinct vulnerabilities arise upon loss of epigenetic silencing or deletion of metabolic genes, such as *BCAT1* and *MTAP*. Meanwhile, it is important to keep in mind that some links between epigenetics and metabolism discussed here may be quite context specific. A key goal lies in understanding how these metabolic-epigenetics links can be exploited therapeutically, particularly in combination strategies.

Additional emerging links between metabolism and epigenetics are likely to also be relevant to cancer cells. Recent work has uncovered a connection between the reactive glycolytic metabolite methylglyoxal and altered chromatin structure due to glycation of histones (Zheng et al. 2019). This modification is detectable in breast cancer tissue and may play a role in cancer pathogenesis in other cancer types. Another emerging area is the discovery of multiple acylation modifications on histones. Since the roles of these modifications in cancer remain poorly understood, they fall outside of the scope of this review but have been discussed in depth in other recent reviews (Sabari et al. 2017, Trefely et al. 2020).

The role of diet in tumorigenesis and in modifying therapeutic responses has also emerged as an important frontier in cancer biology (Bose et al. 2020, Kanarek et al. 2020, Tajan & Vousden 2020). The availability of nutrients that can impact the epigenome, such as methionine, serine, and ascorbate, as well as metabolites such as acetate and butyrate, is directly impacted by dietary composition. Diet and systemic metabolism can modify risk of multiple cancers, although the underlying mechanisms are incompletely understood. Further study into the role of diet in determining tumor metabolic and epigenetic features has the potential to identify distinct vulnerabilities and provide rational strategies to combine nutritional interventions with other therapeutics.

## Figures and Tables

**Table 1:** Epigenetic alterations leading to targetable metabolic vulnerabilities

Genetic alteration/condition	Cancer type	Rationale/epigenetic or metabolic consequence	Proposed therapeutic vulnerability
EZH2 deficiency (Gu et al. 2019)	Leukemia (leukemic-initiating cells)	Decreased histone H3K27me3 and increased BCAT1 expression	mTOR inhibition (rapamycin), BCAT1 inhibition (Gbp)
BAP1 deficiency (Zhang et al. 2018, Liu et al. 2020)	Renal cancer, mesothelioma	Increased H2Aub and increased SLC7A11	GLUT-1 inhibition (KL-11743 or BAY-876)
ARID1A deficiency (Ogiwara et al. 2019)	Colon cancer, ovarian cancer	Decreased expression of SLC7A11	GSH limitation (APR-246 and PRIMA-1)
Tyrosine kinase inhibition (Wang et al. 2019)	Lung adenocarcinoma	Decreased H3K9me2/3 and increased BCAT1 expression	BCAT1 inhibition, ROS inducers (piperlongumine, phenethyl isothiocyanate, auranofin), or GSH synthesis inhibition (BSO)
BRG1 overexpression (Wu et al. 2016)	Breast cancer	Increased fatty acid synthesis gene expression	BRG1 inhibition (ADAADi) and fatty acid synthesis inhibitors (TOFA, C75)
SIRT6 deficiency (Sebastián et al. 2012)	Colorectal carcinoma	Increased glycolytic gene expression	PDK1 inhibition (DCA), potential glycolytic dependency
LSD1 overexpression (Sakamoto et al. 2015)	Hepatocellular carcinoma	Decreased H3K4me2/3, decreased oxidative phosphorylation gene expression	Potential glycolytic dependency
BET inhibition (JQ-1) (Carrer et al. 2019, Sdelci et al. 2019)	Leukemia, lung adenocarcinoma	BRD4 interacts with MTHFD1	Antifolate therapy (methotrexate)
	Pancreatic cancer	Acetyl-CoA metabolic process dependencies	Statins
KMT2D deficiency (Alam et al. 2020)	Lung cancer	Altered superenhancers genome wide, downregulation of *PER2*, increased glycolytic gene expression	2-Deoxyglucose

Abbreviations: acetyl-CoA, acetyl coenzyme A; BSO, buthionine sulfoximine; DCA, dichloroacetate; Gbp, gabapentin; GSH, glutathione; ROS, reactive oxygen species; TOFA, 5-(tetradecyloxy)-2-furoic acid.
